# 

**Published:** 1876-06

**Authors:** 


### CONTENTS:

### ORIGINAL COMMUNICATIONS.

ARTICLE I.—The Teeth and their Preservation. By M. H. Webb, D. D. S.......... 49

ARTICLE II.—Electricity in Dental Surgery. By Geo. H. Perine, D. D. S.......... 62

California State Dental Association.......... 63

The District Dental Society.......... 66

### SELECTED ARTICLES.

ARTICLE III—Artificial Separations of the Teeth as a means of Prevention of Decay By Dr. G. R. Thomas.......... 67

ARTICLE IV—Is the race Degenerating? By John Murray, D. D. S.......... 74

ARTICLE V.—Cylinder Filling. By C. W. Spalding, D. D. S.......... 79

ARTICLE VI.—A New Way of Filling Roots. By H. S. Chase, M. D., D. D. S.... 84

### EDITORIAL, ETC.

Tooth Anomaly, from Dr. S. P. Cutler.......... 85

### MONTHLY SUMMARY.

Bonwill's Method of Inducing Anæsthesia.......... 87
Arrest of Hemorrhage by Torsion.......... 88
A Curious Case of Fracture.......... 89
Female Medical Students.......... 89
The Parasite Theory of Diseases.......... 90
Skull Changes in Rickets.......... 90
New Mode of Obtaining Local Anæsthesia.......... 90
Morphia in Neuralgia.......... 91
Salicylic Acid for Offensive Breath and Expectoration.......... 91
Food for Lean Women.......... 92
Cooking by Cold.......... 92
Hardening Compound for Steel.......... 93
Radical Cure for Piles.......... 93
Painful Points in Neuralgia.......... 93
Jaborandi in Colds.......... 93
Torsion of Arteries.......... 93
Exposure of Spiritualism.......... 94
Gelseminum Sempervireus in Facial Neuralgia.......... 95
A Lotion for Fetid Feet and Arm Pits.......... 95
Sensitive Artificial Eyes.......... 95
Cure for Corns.......... 96
A Large Mouth.......... 96
Graduates in Dentistry.......... 96
Johns Hopkins Hospital.......... 96
Eucalyptus in Ague.......... 96

